# Monotherapy with a low-dose lipopeptide HIV fusion inhibitor maintains long-term viral suppression in rhesus macaques

**DOI:** 10.1371/journal.ppat.1007552

**Published:** 2019-02-04

**Authors:** Huihui Chong, Jing Xue, Yuanmei Zhu, Zhe Cong, Ting Chen, Qiang Wei, Chuan Qin, Yuxian He

**Affiliations:** 1 MOH Key Laboratory of Systems Biology of Pathogens, Institute of Pathogen Biology and Center for AIDS Research, Chinese Academy of Medical Sciences and Peking Union Medical College, Beijing, China; 2 Key Laboratory of Human Disease Comparative Medicine, Chinese Ministry of Health, Beijing Key Laboratory for Animal Models of Emerging and Remerging Infectious Diseases, Institute of Laboratory Animal Science, Chinese Academy of Medical Sciences and Comparative Medicine Center, Peking Union Medical College, Beijing, China; Emory University, UNITED STATES

## Abstract

Combination antiretroviral therapy (cART) dramatically improves survival of HIV-infected patients, but lifelong treatment can ultimately result in cumulative toxicities and drug resistance, thus necessitating the development of new drugs with significantly improved pharmaceutical profiles. We recently found that the fusion inhibitor T-20 (enfuvirtide)-based lipopeptides possess dramatically increased anti-HIV activity. Herein, a group of novel lipopeptides were designed with different lengths of fatty acids, identifying a stearic acid-modified lipopeptide (LP-80) with the most potent anti-HIV activity. It inhibited a large panel of divergent HIV subtypes with a mean IC_50_ in the extremely low picomolar range, being > 5,300-fold more active than T-20 and the neutralizing antibody VRC01. It also sustained the potent activity against T-20-resistant mutants and exhibited very high therapeutic selectivity index. Pharmacokinetics of LP-80 in rats and monkeys verified its potent and long-acting anti-HIV activity. In the monkey, subcutaneous administration of 3 mg/kg LP-80 yielded serum concentrations of 1,147 ng/ml after injection 72 h and 9 ng/ml after injection 168 h (7 days), equivalent to 42,062- and 330-fold higher than the measured IC_50_ value. In SHIV infected rhesus macaques, a single low-dose LP-80 (3 mg/kg) sharply reduced viral loads to below the limitation of detection, and twice-weekly monotherapy could maintain long-term viral suppression.

## Author summary

T-20 is the only clinically approved viral fusion inhibitor, which is used in combination therapy for HIV-1 infection; however, it exhibits relatively low antiviral activity and easily induces drug resistance. Here we report a lipopeptide fusion inhibitor termed LP-80, which exhibits the most potent activity in inhibiting divergent HIV-1 subtypes. Especially, LP-80 has extremely potent and long-acting therapeutic efficacy with very low cytotoxicity, making it an ideal drug candidate for clinical use. Furthermore, LP-80 and its truncated versions can be used as important probes for exploiting the mechanisms of viral fusion and inhibition.

## Introduction

Six classes of anti-HIV drugs block different steps of the viral life cycle, including cell entry, reverse transcription, integration and virion maturation [1]. Highly active antiretroviral therapy (HAART) with multiple drugs in a combination can suppress the virus to below the limitation of detection, thus leading to profound reductions in morbidity and mortality associated with AIDS. Because of the lack of an effective vaccine, antiretroviral therapy has also been considered a vital strategy to control the HIV transmission. Different from other drugs that act after infection occurs, HIV entry inhibitors intercept the virus before it invades the target cells. Currently, there are two anti-HIV drugs targeting the entry process: while maraviroc binds to the coreceptor CCR5 thus being used to treat infections by CCR5-tropic HIV isolates, the peptide drug enfuvirtide (T-20) acts by blocking the fusion between viral and cell membranes [2–4]. T-20 is effective in combination therapy, but it exhibits relatively weak anti-HIV activity, short half-life, and low genetic barrier to inducing drug resistance [5,6], calling for new membrane fusion inhibitors with improved pharmaceutical profiles.

Emerging studies demonstrate that lipid conjugation is a more efficient strategy for designing peptide inhibitors that target the viral fusion step [7–12]. So-called lipopeptides can anchor to the target cell membranes thereby raising the concentrations of the inhibitors at the viral entry site [7,11]. In sequence structure, T-20 has a C-terminal tryptophan-rich motif (TRM), which is considered a membrane-binding domain (LBD) that can interact with the target cell membrane to confer antiviral activity [13–15]. By substituting the TRM of T-20 with C16 fatty acid (palmitic acid), we previously generated the lipopeptide LP-40, which showed significantly increased anti-HIV activity [16]. The potency of LP-40 could be dramatically improved by introducing the intrahelical salt-bridge-prone and HIV-2/SIV sequences, as evidenced by LP-50 and LP-52 that inhibited divergent HIV-1 isolates at very low picomolar (pM) concentrations [17,18]. It is known that the fatty acid length, polarity and bulkiness can critically determine the pharmacokinetic of a peptide inhibitor [12,13,19,20]. For example, a long-acting glucagon-like peptide-1 derivative for type 2 diabetes has been successfully developed by replacing C16 with C18 (stearic acid): while C16-conjugated liraglutide requires an once-daily dosage, C18-conjugated semaglutide is used once-weekly [21,22]. In order to develop a more efficient HIV fusion inhibitor for clinical development, herein we generated and characterized a panel of new lipopeptides with various fatty acids, including C18, C8 (octanoic acid), C12 (lauric acid), C20 (arachidic acid), C22 (docosanoic acid), and C24 (lignoceric acid). It was found that C18-conjugated lipopeptide LP-80 had the most potent anti-HIV activity and showed a long-acting therapeutic efficacy in SHIV-infected rhesus monkey models.

## Results

### Generation of novel HIV fusion inhibitors with diverse fatty acids

**Fig 1:**
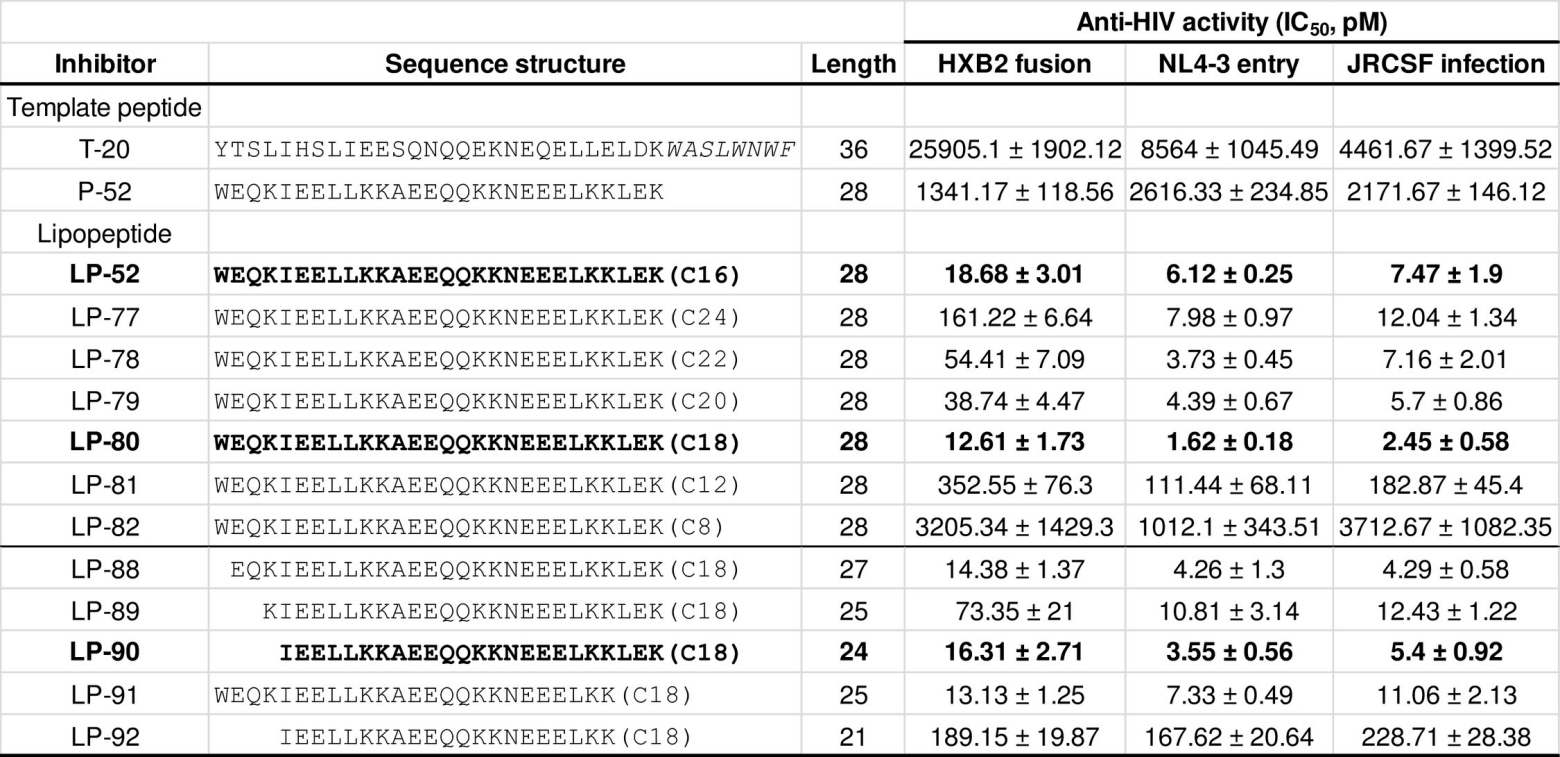
Structural and functional characterization of fatty acid-conjugated HIV fusion inhibitors. The inhibitory activities of inhibitors on HIV-1_HXB2_ Env-mediated cell-cell fusion, HIV-1_NL4-3(D36G)_ pseudovirus entry, and HIV-1_JRCSF_ infection were respectively determined. The TRM sequence of T-20 is on italic. C16, palmitic acid; C24, lignoceric acid; C22, docosanoic acid; C20, arachidic acid; C18, stearic acid; C12, lauric acid; C8, octanoic acid). The antiviral experiments were performed in triplicate and repeated three times. The data of three assays are expressed as means ± standard deviations (SD).

We recently identified that the chimeric peptide P-52 is an ideal template for design of lipopeptide-based fusion inhibitors with high activities against HIV-1, HIV-2, and SIV isolates [17]. In this study, we focused on examining the significance of the fatty acid carbon chain length in the development of a more efficient anti-HIV drug. P-52 was conjugated with different lengths of fatty acids, resulting in a group of new lipopeptides termed LP-77 (C24), LP-78 (C22), LP-79 (C20), LP-80 (C18), LP-81 (C12), and LP-82 (C8). Then, the anti-HIV activities of diverse lipopeptides were determined with three functional approaches. As shown in Fig 1, LP-80 exhibited the most potent activities in inhibiting HIV-1_HXB2_ Env-mediated cell-cell fusion, HIV-1_NL4-3_ pseudovirus-mediated single-cycle cell entry, and replication-competent HIV-1_JRCSF_-mediated infection, with mean 50% inhibitory concentrations (IC_50_s) of 12.61, 1.62, and 2.45 pM, respectively. In comparison, LP-52 and the lipopeptides with longer fatty acids (LP-77, LP-78, and LP-79) showed relatively lower anti-HIV activities, and the lipopeptides with shorter fatty acids (LP-81 and LP-82) had markedly decreased potencies. Furthermore, a group of truncated lipopeptides were produced by referring the sequence of C18-conjugated LP-80, and their inhibitory activities were characterized. It was found that the N-terminally (LP-88, LP-89, LP-90) or C-terminally (LP-91) truncated inhibitors still sustained very high anti-HIV potencies, especially LP-90 (24-mer) which inhibited HIV-1_HXB2_, HIV-1_NL4-3,_ and HIV-1_JRCSF_ with IC_50_s of 16.31, 3.55, and 5.4 pM, respectively. Consistent with our previous observation on C16-conjugated lipopeptides [17], addition of an N-terminal lysine residue seemly exerted a negative effect on the activity of LP-89. Truncation from both the N- and C-terminals of LP-80 resulted in LP-92, which only had a 21-amino acid core sequence and showed greatly reduced antiviral activity; however, LP-92 remained a highly active HIV fusion inhibitor relative to T-20.

### Structural properties and binding stability of new lipopeptide inhibitors

**Fig 2:**
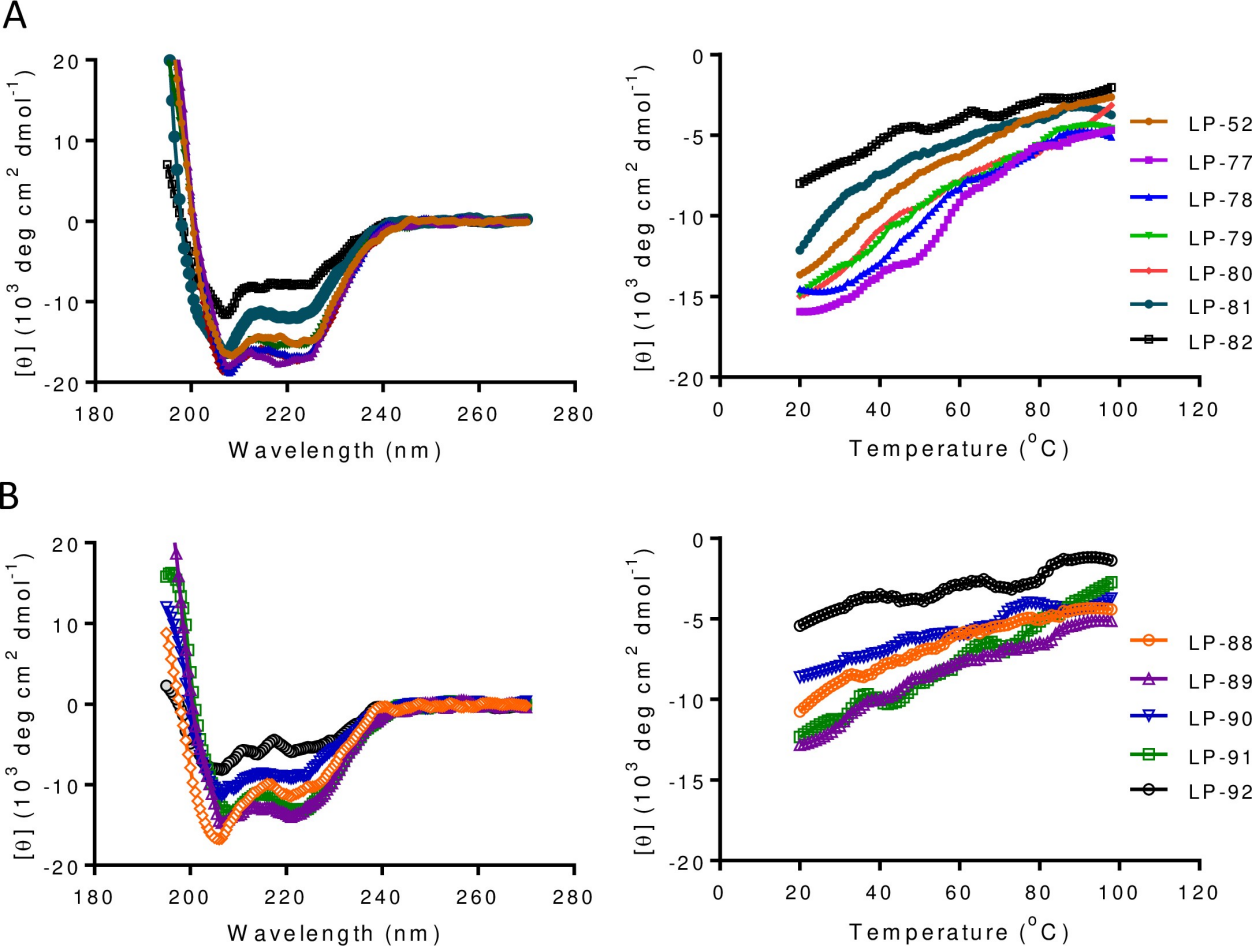
Secondary structure and stability of diverse fatty acid-modified HIV fusion inhibitors determined by CD spectroscopy. **(A)** The α-helicity (left) and thermostability (right) of diverse fatty acid-modified inhibitors. **(B)** The α-helicity (left) and thermostability (right) of truncated C18-conjugated lipopeptides. The final concentration of each peptide in PBS was 10 μM. The experiments were repeated two times, and representative data are shown.

**Fig 3:**
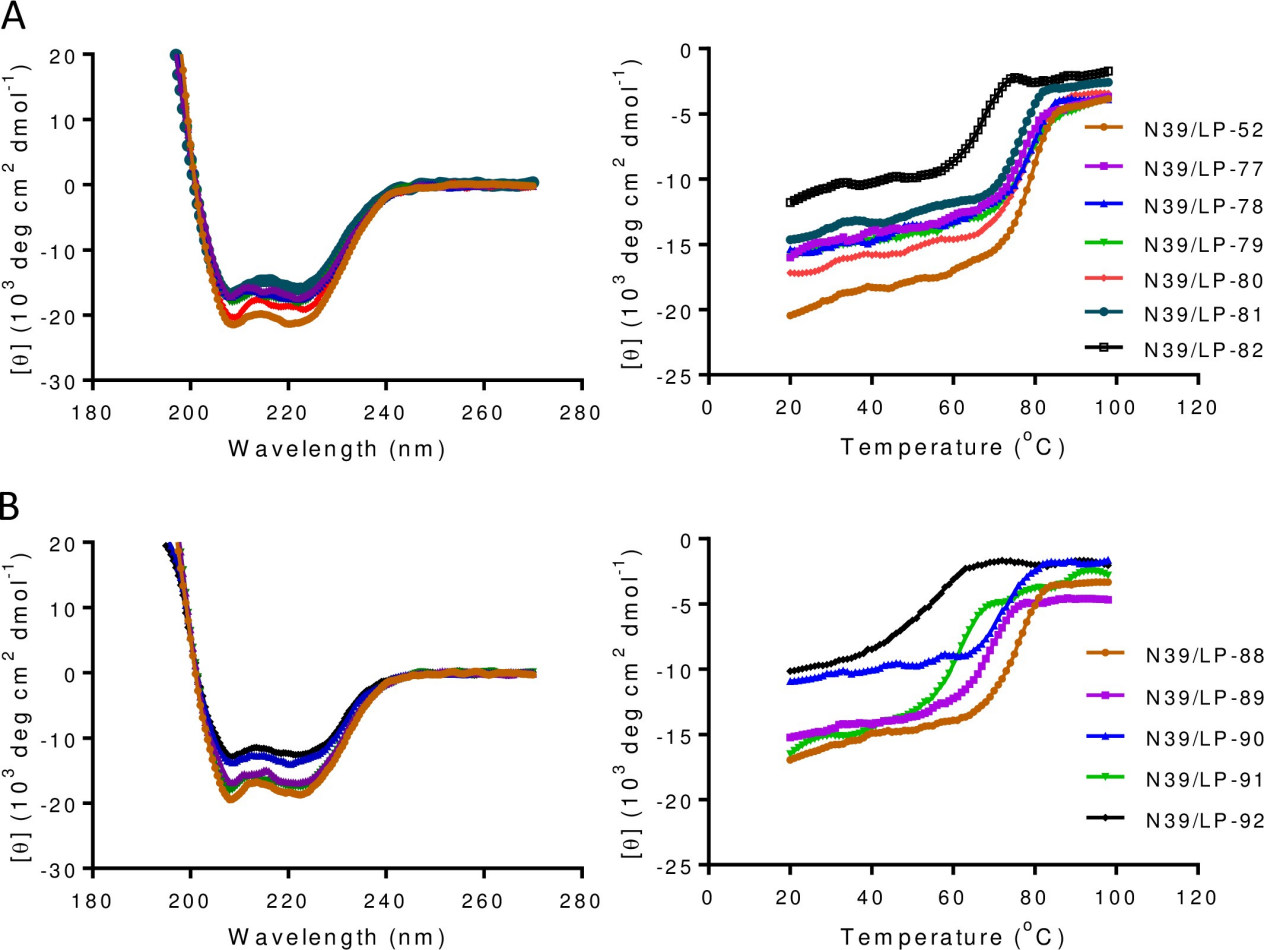
Interaction of lipopeptide inhibitors with a target mimic peptide determined by CD spectroscopy. **(A)** The α-helicity (left) and thermostability (right) of diverse fatty acid-modified inhibitors in complexes with the gp41 NHR-derived target mimic peptide N39. **(B)** The α-helicity (left) and thermostability (right) of truncated C18-conjugated lipopeptides in complexes with N39. The final concentration of each peptide in PBS was 10 μM. The experiments were repeated two times, and representative data are shown.

To further explore the structure and activity relationship (SAR) of fatty acid-conjugated lipopeptides, we used circular dichroism (CD) spectroscopy to analyze their secondary structures and thermostabilities. As shown in Fig 2 and Table 1, C16, C18, C20, C22, and C24-conjugated lipopeptides (LP-52, LP-80, LP-79, LP-78, and LP-77) had comparable α-helicity and thermostability, but both of which reduced in C8 and C12-conjugated lipopeptides (LP-82 and LP-81). Similarly, all the truncated C18-conjugated lipopeptides (LP-88 ~ LP-92) showed reduced α-helicity and thermostability. Next, we analyzed the interactions of diverse lipopeptides with an NHR-derived target mimic peptide (N39). As shown Fig 3 and Table 1, all the inhibitors interacted with N39 to display increased α-helical contents. As compared to C16-conjugated LP-52, the longer fatty acids did not obviously affect the α-helicity and thermostability of the lipopeptides, as shown by LP-77, LP-78, LP-79, and LP-80; in contrast, conjugation with the shorter fatty acids resulted in greatly decreased α-helical contents and *T*_m_ values, as shown by LP-81 and LP-82. Consistently, all the truncated lipopeptides exhibited a markedly decreased binding stability either. As suggested by LP-90 and LP-91, while the N-terminal WEQK motif critically determined the α-helicity, the C-terminal LEK motif was more important in the binding stability. Taken together, these results demonstrated that both the fatty acid length and amino acid sequence are critical determinants for the conformation and stability of lipopeptide inhibitors.

**Table 1:** Structural properties and binding stabilities of diverse lipopeptide fusion inhibitors^a^.

Lipopeptide	Fatty acid	[θ]_222_	Helix content (%)	*T*_m_ (°C)
LP-52	C16	-14952	45	56
LP-77	C24	-16571	50	56
LP-78	C22	-16798	51	57
LP-79	C20	-15935	48	56
LP-80	C18	-17025	52	56
LP-81	C12	-12161	37	NA
LP-82	C8	-7365	22	NA
LP-88	C18	-11066	34	NA
LP-89	C18	-14238	43	NA
LP-90	C18	-9925	30	NA
LP-91	C18	-13779	42	NA
LP-92	C18	-5119	16	NA
N39/LP-52	C16	-21060	64	79
N39/LP-77	C24	-17985	55	77
N39/LP-78	C22	-17209	52	77
N39/LP-79	C20	-18030	55	78
N39/LP-80	C18	-18955	57	79
N39/LP-81	C12	-16313	49	75
N39/LP-82	C8	-12910	39	65
N39/LP-88	C18	-18242	55	76
N39/LP-89	C18	-16781	51	70
N39/LP-90	C18	-13989	42	72
N39/LP-91	C18	-18156	55	61
N39/LP-92	C18	-12476	38	55

^a^The assay was performed 2 times, and representative data are shown.

### LP-80 and LP-90 are extremely potent inhibitors of divergent HIV-1 subtypes

As demonstrated above, the C18-conjuagted lipopeptide LP-80 had highest antiviral and target-binding activities. Similar to the C16-conjugated lipopeptides [17], the N-terminally truncated version LP-90 manifested very high capacities either. Herein, we sought to validate the inhibitory activities of LP-80 and LP-90 with HIV-1 isolates possessing different genotypes and phenotypes. First, six replication-competent HIV-1 strains were applied. As shown in Table 2, the control inhibitors T-20 and LP-52 inhibited six viruses with IC_50_s of 15,305.37 and 16.95 pM, respectively, whereas LP-80 and LP-90 had their IC_50_s of 7.09 and 23.71 pM, respectively. Furthermore, a large panel of pseudoviruses with their Envs derived from primary HIV-1 isolates were prepared and used to determine the antiviral activities of the inhibitors by a single-cycle infection assay. Apart from T-20 and LP-52, the previously characterized fusion inhibitor C34-Chol and broadly neutralizing antibody VRC01 were also tested for comparison [7,23]. As shown in Table 3, T-20, LP-52, LP-80, LP-90, C34-Chol, and VRC01 inhibited divergent HIV-1 subtypes with mean IC_50_s of 29,950.09, 16.97, 5.58, 13.38, 68.06, and 33382.09 pM, respectively. Therefore, the antiviral activity of LP-80 was about 5367-, 3-, 12-, and 5982-folds higher than that of T-20, LP-52, C34-Chol, and VRC01, respectively.

**Table 2:** Inhibitory activities of fusion inhibitors against replication-competent HIV-1 isolates^a^.

			Mean IC_50_ (pM) ± SD			
HIV-1	Subtype	Tropism	T-20	LP-52	LP-80	LP-90
NL4-3	B	X4	47386.67 ± 4815.83	4.5 ± 2.49	2.45 ± 0.51	76.78 ± 19.02
LAI	B	X4	1163.33 ± 102.94	12.22 ± 0.93	5.85 ± 0.86	8.34 ± 1.54
SG3	B	X4	2851.32 ± 226.64	5.22 ± 1.89	1.8 ± 0.42	1.67± 0.61
JR-CSF	B	R5	2968.33 ± 788.34	8.02 ± 3.59	4.02 ± 2.63	9.33 ± 1.97
WITO.c/2474	B	R5	7810.56 ± 1624.26	5.29 ± 1.61	3.38 ± 1.09	4.06 ± 1.67
RHPA.c/2635	B	R5	6517.44 ± 1329.99	9.42 ± 1.34	4.81 ± 1.98	5.36 ± 1.66
THRO.c/2626	B	R5	31310 ± 2387.75	27.12 ± 4.61	14.93 ± 1.93	14.59 ± 2.24
CH040.c/2625	B	R5	30883.33 ± 3805.27	66.67 ± 9.1	22.67 ± 5.55	50.18 ± 13.77
89.6	B	R5X4	15431.01 ± 5882.09	17.49 ± 2.05	5.27 ± 0.3	57.44 ± 10.92
R3A	B	R5X4	6731.67 ± 1082.18	13.58 ± 1.99	5.67 ± 0.85	9.32 ± 1.21
**Mean**			**15305.37**	**16.95**	**7.09**	**23.71**

^a^The assay was performed in triplicate and repeated 3 times. The data of all 3 assays are expressed as means ± standard deviations (SD).

**Table 3:** Inhibitory activities of fusion inhibitors against divergent HIV-1 subtypes^a^.

		Mean IC_50_ (pM) ± SD					
Primary Env	Subtype	T-20	LP-52	LP-80	LP-90	C34-Chol	VRC01
92UG037.8	A	7760.33 ± 1035.74	28.19 ± 8.27	3.72 ± 1	8.42 ± 1.52	97.37 ± 17.52	1708.4 ± 510.92
92RW020	A	3243 ± 231.51	39.33 ± 1.51	9.74 ± 0.93	15.1 ± 0.62	96.57 ± 5.5	1255.28 ± 216.49
398-F1_F6_20	A	19386.67 ± 1711.2	9.48 ± 1.74	1.49 ± 0.26	6.32 ± 0.46	15.72 ± 0.86	2063.55 ± 666.79
PVO	B	69691.67 ± 697.56	29.68 ± 7.83	12.97 ± 1.67	22.62 ± 4.7	96.54 ± 8.99	5811.01 ± 441.64
pREJO4541	B	54978.67 ± 2900.09	7.22 ± 0.85	1.4 ± 0.61	15.95 ± 3.13	55.35 ± 9.24	734.8 ± 173.19
SF162	B	17579 ± 1032.38	26.38 ± 3.81	3.12 ± 0.37	4.82 ± 0.91	35.07 ± 1.24	416.38 ± 69.28
JRFL	B	9622 ± 502.49	46.68 ± 6.96	10.45 ± 2.25	12.2 ± 0.83	126.4 ± 9	349.03 ± 25.98
SC422661.8	B	16102.67 ± 2243.99	9.39 ± 1.76	2.55 ± 0.77	38.53 ± 7.05	88.63 ± 10.89	838.89 ± 147.21
AC10.0.29	B	2845 ± 102.56	9.71 ± 0.81	5.01 ± 0.93	4.42 ± 0.28	37.24 ± 5.72	15332.74 ± 381.02
TRO.11	B	7048.33 ± 1334.84	20.6 ± 2.67	3.92 ± 0.26	8.95 ± 2.3	37.28 ± 1.78	789.91 ± 25.98
X2278_C2_B6	B	5660.33 ± 74.44	1.17 ± 0.22	0.27 ± 0.01	1.04 ± 0.22	8.41 ± 1.69	942.99 ± 259.79
R3A	B	6879.67 ± 1121.69	9.22 ± 1.9	6.34 ± 0.65	9.32 ± 1.21	54.11 ± 9.93	21186.62 ± 2571.78
B01	B‘	84448.67 ± 3693.05	7.06 ± 1.16	3.49 ± 0.4	17.95 ± 1.58	46.26 ± 9.21	23684.92 ± 5161.15
B02	B'	10362 ± 529.34	14.94 ± 1.55	8.9 ± 1.03	15.53 ± 3.68	53.99 ± 11.56	281.67 ± 17.32
B04	B'	5647.67 ± 366.49	13.36 ± 0.94	3.81 ± 1.08	17.49 ± 7.72	77.87 ± 11.45	60932.96 ± 48242.93
43–22	B‘	23217 ± 592.5	7.83 ± 0.74	3.24 ± 0.27	3.9 ± 1.33	31.47 ± 5.16	391.89 ± 17.32
Du156	C	15744.67 ± 1578.06	4.66 ± 0.75	1.1 ± 0.35	3.63 ± 0.67	28.02 ± 3.74	685.81 ± 69.28
ZM53M.PB12	C	22727.33 ± 943.43	26.85 ± 3.04	8.62 ± 0.74	8.81 ± 0.46	90.51 ± 10.56	4176.09 ± 363.71
CAP210.2.00.E8	C	123120.33 ± 4772.77	39.25 ± 3.57	10.61 ± 2.56	29.07 ± 1.33	86.53 ± 15.24	>300000
CAP45.2.00.G3	C	13608.67 ± 2044.57	7.58 ± 0.64	1.69 ± 0.48	2.14 ± 0.13	14.03 ± 1.38	35643.73 ± 37071.98
CE703010217_B6	C	41837 ± 2215.85	11.65 ± 1.37	5.72 ± 1.32	11.17 ± 0.83	92.8 ± 10.3	2877.95 ± 259.79
HIV_25710–2.43	C	13727.67 ± 1854.12	14.13 ± 1.91	3.2 ± 0.68	16.02 ± 2.93	58.95 ± 8.54	10164.68 ± 692.77
CE1176_A3	C	8398.67 ± 495.68	11.06 ± 1.52	2.94 ± 0.19	8.77 ± 1.95	89.22 ± 3.26	16551.28 ± 891.94
X1632-S2-B10	G	15552.67 ± 2008.19	24.54 ± 2.39	5.56 ± 0.76	28.37 ± 4.11	112.47 ± 9.06	838.89 ± 147.21
246_F3_C10_2	A/C	36174 ± 3699.13	17.48 ± 1.91	4.05 ± 0.47	6.84 ± 0.47	39.92 ± 4.19	8401.17 ± 450.3
AE03	A/E	12379.33 ± 109.25	10.87 ± 1.89	3.44 ± 0.83	14.06 ± 4.17	19.95 ± 5.35	55.11 ± 25.98
GX11.13	A/E	24897.33 ± 2214.12	8.7 ± 0.88	4.85 ± 0.99	24.81 ± 1.59	146.1 ± 19.48	11799.6 ± 1151.73
SHX335.24	A/E	40605 ± 1424.96	18.87 ± 0.34	2.83 ± 0.66	3.01 ± 0.2	52.61 ± 2.87	2553.42 ± 25.98
CNE8	A/E	24788.67 ± 1877.99	4.97 ± 1.31	3.56 ± 0.7	22.52 ± 2.6	125.1 ± 5.36	7733.73 ± 666.79
CNE55	A/E	25136.33 ± 2332.46	17.31 ± 1.41	1.11 ± 0.18	2.54 ± 0.89	47.36 ± 9.1	4280.19 ± 164.53
CH64.20	B/C	33294 ± 1176.85	9.71 ± 0.84	5.75 ± 0.19	7.72 ± 2.67	43.13 ± 1.94	6711.14 ± 207.83
CH070.1	B/C	164753 ± 28325.58	27.6 ± 3.33	28.51 ± 0.63	31.73 ± 2.04	158.17 ± 21.86	282039.19 ± 4503.02
CH110	B/C	31005.33 ± 2665.34	7.98 ± 1.3	3.3 ± 0.29	7.85 ± 1.22	39.89 ± 1.18	14561.21 ± 4173.95
CH119.10	B/C	11514.33 ± 1747.02	10.65 ± 1.62	4.7 ± 0.35	10.79 ± 2.36	48.3 ± 4.58	55115.82 ± 3455.2
CH120.6	B/C	48146.33 ± 4919.64	19.88 ± 1.48	10.55 ± 1.02	25.4 ± 1.62	119.5 ± 3.52	845.02 ± 155.87
BJOX002000.03.2	B/C	26320 ± 2766.81	36.77 ± 2.93	8.48 ± 0.08	13.91 ± 2.46	79.29 ± 7.54	>300000
**Mean**		**29950.09**	**16.97**	**5.58**	**13.38**	**68.06**	**33382.09**

^a^The assay was performed in triplicate and repeated 3 times. The data of all 3 assays are expressed as means ± SD.

### LP-80 sustains its potent activity against T-20-resistant mutants

To exploit the mechanism of action of LP-80, we also performed the *in vitro* selection of HIV-1 mutants resistant to LP-80. So far the experiment failed because it was difficult to passage the virus in the presence of LP-80 inhibitor; by contrast, the concentration of the control peptide T-20 could be easily raised to 10,000 nM. Alternatively, we prepared a panel of HIV-1_NL4-3_ Env-based T-20-resistant mutants and examined the inhibitory activities of LP-80 and LP-90 by the single-cycle infection assay. As shown in Table 4, both LP-80 and LP-90 displayed markedly reduced activities against diverse HIV-1_NL4-3_ mutant viruses, verifying that they also targeted the NHR sites of gp41 that critically determined the cross-resistance. Despite this, LP-80 and LP-90 remained highly potent inhibitors of T-20-resistant mutants, which might reflect their relatively higher genetic barriers to inducing resistance. By comparing the fold IC_50_ changes by LP-80 and LP-90, the data also verified the importance of the N-terminal WEQK motif of the newly-designed lipopeptide inhibitors in overcoming the T-20 resistance problem, consistent with our previous findings [17].

**Table 4:** Inhibitory activities of LP-80 and LP-90 on T-20-resistant HIV-1 mutants^a^.

	T-20		LP-80		LP-90	
Pseudovirus	IC_50_ (nM)	Fold change	IC_50_ (nM)	Fold change	IC_50_ (nM)	Fold change
**T-20 sensitive**						
NL4-3_D36G_	8.77 ± 1.02	1	<0.01 (0.002)	1	<0.01 (0.003)	1
**T-20 resistant**						
NL4-3_WT_	165.83 ± 18.99	18.91	<0.01 (0.002)	1	0.03 ± 0	10
NL4-3_I37T_	1222.89 ± 119.72	139.44	0.05 ± 0	25	0.51 ± 0.09	170
NL4-3_V38A_	2239.78 ± 33.11	255.39	0.23 ± 0.03	115	8.25 ± 0.55	2750
NL4-3_V38M_	1054.12 ± 74.83	120.2	0.13 ± 0.02	65	4.32 ± 0.78	1440
NL4-3_Q40H_	1148.89 ± 87.97	131	0.13 ± 0.02	65	4.17 ± 0.14	1390
NL4-3_N43K_	748.49 ± 41.88	85.35	0.25 ± 0.02	125	5.05 ± 0.13	1683.33
NL4-3_G36S/V38M_	317.26 ± 1.65	36.18	0.07 ± 0.01	35	2.37 ± 0.39	790
NL4-3_I37T/N43K_	6950.33 ± 1144.95	792.51	3.79 ± 0.05	1895	46.33 ± 0.79	15443.33
NL4-3_V38A/N42T_	5954.11 ± 699.55	678.92	1.25 ± 0.01	625	49.19 ± 2.19	16396.67

^a^The assay was performed in triplicate and repeated 3 times. The data of all 3 assays are expressed as means ± SD.

### LP-80 exhibits extremely low cytotoxicity

**Fig 4:**
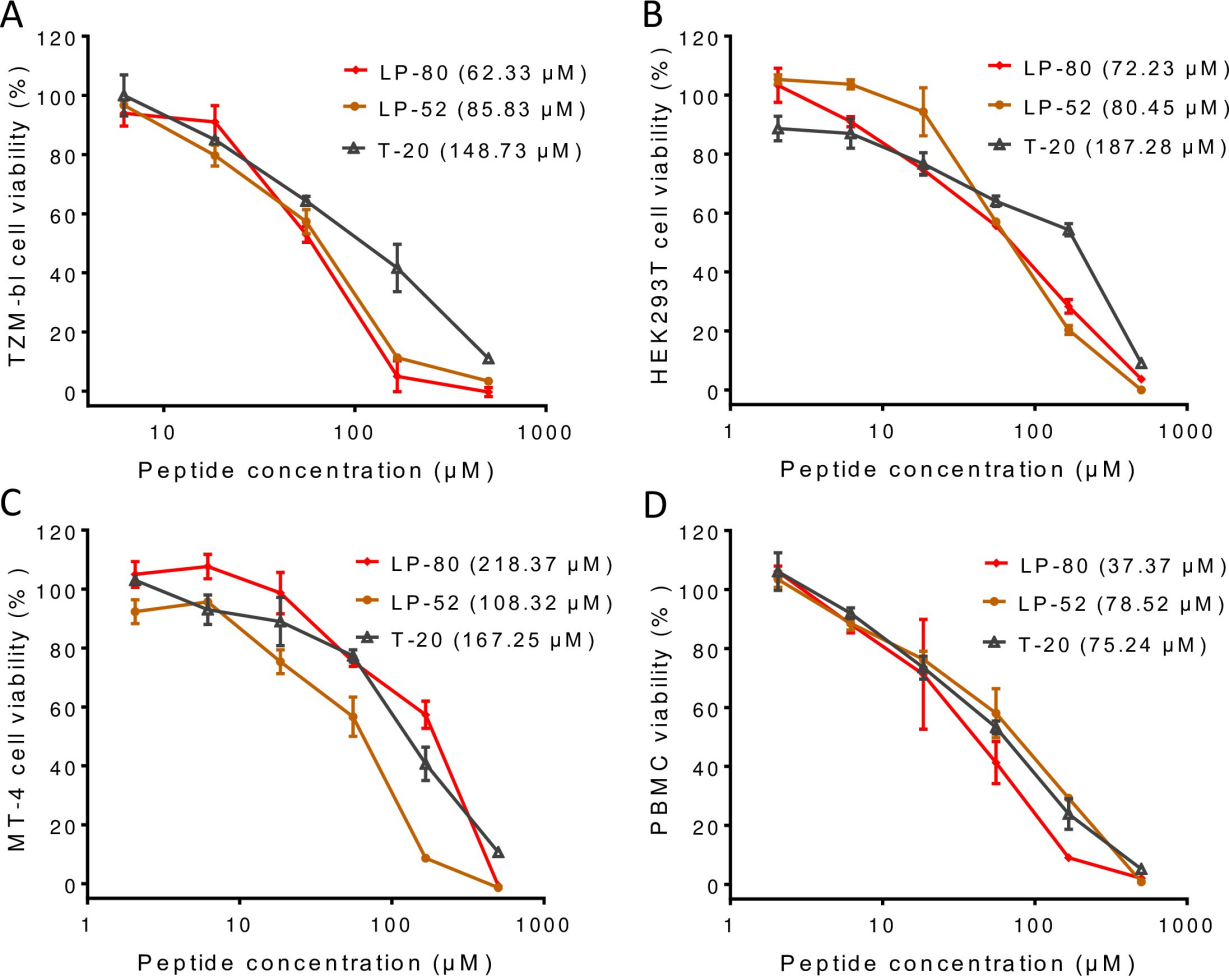
Cytotoxicity of LP-80 in comparison with T-20 and LP-52. The cytotoxicity of inhibitors on TZM-bl **(A)**, HEK293T **(B)**, MT-4 **(C)**, and human PBMC **(D)** was measured by a CellTiter 96 AQ_ueous_ One Solution cell proliferation assay. 50% cytotoxic concentrations (CC_50_s) were calculated and are presented in parentheses.

We previously reported that T-20 derivatives, such as LP-50, LP-51, and LP-52, possess extremely low cytotoxicity and high genetic resistance barriers [17,18]. In this study, we also determined the cytotoxicity of LP-80 in three different cell lines and human peripheral blood mononuclear cells (PBMCs). As shown in Fig 4, LP-80 had a 50% cytotoxic concentration (CC_50_) of 62.33 μM in TZM-bl cells, of 72.23 μM in HEK293T cells, of 218.37 μM in MT-4 cells, and of 37.37 μM in human PBMCs, which were comparable with that of T-20 and LP-52. Considering their antiviral activities at very low picomolar concentrations, the presented results suggested again that fatty acid-based lipopeptide fusion inhibitors possess an extremely high therapeutic selectivity index (CC_50_/IC_50_ ratio).

### Pharmacokinetics of LP-80 in rats and rhesus monkeys

**Fig 5:**
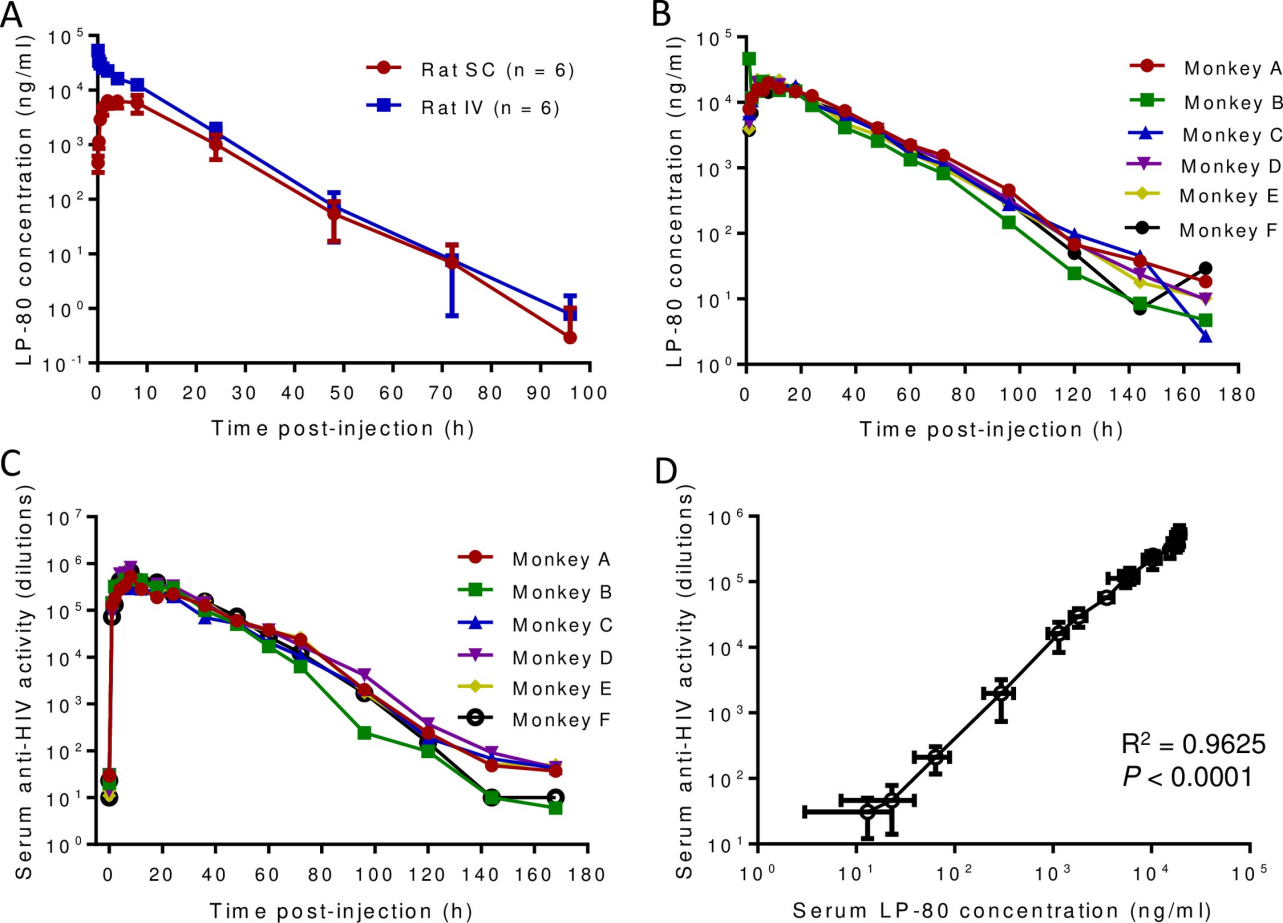
Pharmacokinetics of LP-80 in rats and rhesus monkeys. **(A)** Pharmacokinetics of LP-80 in rats. LP-80 was subcutaneously (SC) or intravenously (IV) injected to rats (n = 6) at 6 mg/kg. **(B)** Pharmacokinetics of LP-80 in monkeys. LP-80 was subcutaneously injected to rhesus monkeys (n = 6) at 3 mg/kg. The concentrations of LP-80 in the serum samples of rat and monkeys were monitored with time. The calculated pharmacokinetic parameters are given in Table 5. **(C)** The inhibitory activities of sera from monkeys administrated LP-80. The fold serum dilution that inhibited 50% of HIV-1_NL4-3_ infection was determined by a single-cycle infection assay. **(D)** Correlation between the serum concentration of LP-80 and its *ex vivo* anti-HIV activity analyzed by Pearson Correlation Coefficient.

To exploit the *in vivo* stability of LP-80, we sought to investigate its pharmacokinetics. First, LP-80 was subcutaneously or intravenously injected into six rats at 6 mg/kg of body weight and its serum concentration was monitored with time. As shown in Fig 5A and Table 5, the subcutaneously injected LP-80 achieved a mean maximum concentration (C_max_) of 7,647 ng/ml with a *T*_1/2_ of 6.28 h; the intravenously injected LP-80 achieved a mean serum C_max_ of 53,259 ng/ml with a *T*_1/2_ of 6.04 h. Impressively, about 7 ng/ml of LP-80 were still detectable in the sera of rats after injection 72 h, which were 257-fold higher than the concentration corresponding to the mean IC_50_ value measured against ten replication-competent viruses (7.09pM, Table 1).

**Table 5:** Pharmacokinetic parameters of LP-80 in rats and rhesus monkeys.

Parameter	Unit	Rat SC (n = 6)	Rat IV (n = 6)	Monkey SC (n = 6)
*T*_max_	h	4 ± 2.19	0.08 ± 0	5.83 ± 3.82
*C*_max_	ng/ml	7647.45 ± 1579.54	53259.22 ± 10579.13	24781.29 ± 10838.97
*t*_1/2_	h	6.28 ± 1.03	6.04 ± 0.85	13.74 ± 0.86
AUC_last_/AUC_0-168h_	h*ng/ml	113862.55 ± 33590.25	288014.36 ± 49430.78	601201.4 ± 29250.77
AUCINF_obs	h*ng/ml	113893.71 ± 33583.35	288037.63 ± 49436.43	601456.28 ± 29272.59
Vz_F_obs/Vz_obs	ml/kg	535.14 ± 250.96	183.41 ± 21.57	98.88 ± 3.4
Cl_F_obs/Cl_obs	ml/h/kg	57.47 ± 20.45	21.31 ± 3.38	5 ± 0.25
MRT_last_	h	9.73 ± 1.11	7.53 ± 0.85	23.68 ± 2.26

Next, we determined the pharmacokinetics of LP-80 in non-human primates. As shown in Fig 5B and Table 5, LP-80 achieved a mean C_max_ of 24,781 ng/ml with a *T*_1/2_ of 13.74 h when injected subcutaneously into six healthy rhesus monkeys at a concentration of 3 mg/kg. LP-80 was still detectable at a mean concentration of 1,147 ng/ml in the sera of monkeys after injection 72 h, which was 42,062-fold higher than the IC_50_ value 7.09 pM, and it sustained at 9 ng/ml after injection 168 h (7 days). To elucidate the relationship between the serum concentration and anti-HIV activity of LP-80, we also measured the inhibitory activity of the monkey sera. As shown in Fig 5C, all the sera showed highly potent and long-lasting activities in inhibiting HIV-1_NL4-3_ infection, with the peak levels during 6–8 h after injection. In line with their concentrations, the sera could inhibit 50% virus infection with mean dilutions of 16,157-fold after 72 h, 1,980-fold after 96 h, 211-fold after 120 h and 31-fold after 168 h. Analyzed by Pearson Correlation Coefficient (Fig 5D), the serum concentration of LP-80 was highly correlated with its *ex vivo* anti-HIV activity (R^2^ = 0.9625, *P* < 0.0001).

### High therapeutic efficacy of LP-80 in chronic SHIV infection

**Fig 6:**
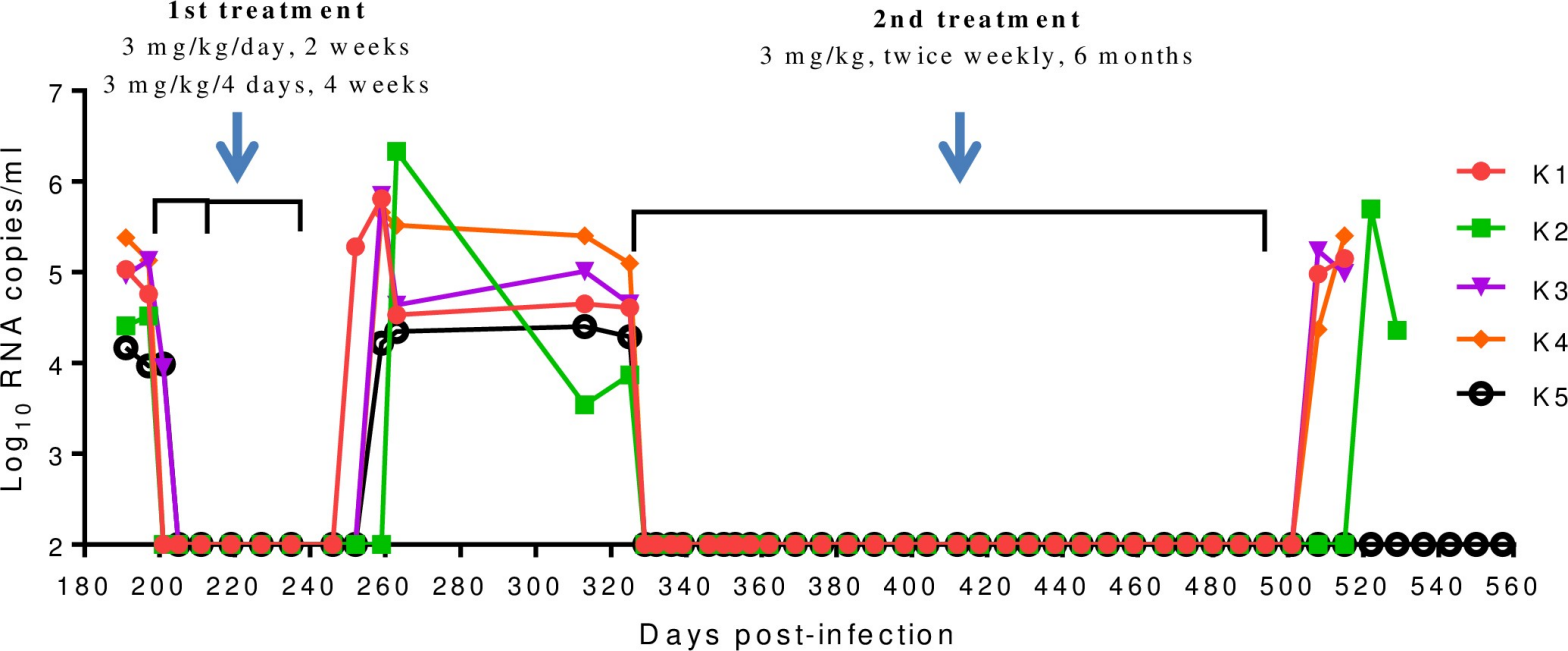
Therapeutic efficacy of LP-80 in chronic SHIV infection. Five Chinese rhesus monkeys (K1 to K5) were chronically infected with SHIV_SF162P3_ over 6 months, and were subcutaneously treated with LP-80 for two rounds. In the 1^st^ treatment, LP-80 was injected at 3 mg/kg, once daily for 2 weeks and followed by once every 4 days for 4 weeks; in the 2^nd^ treatment, LP-80 was injected at 3 mg/kg, twice weekly for 6 months. Plasma viral RNA loads (copies/ml) were measured by quantitative RT-PCR with a detection limit of 100 copies/ml.

We further investigated the therapeutic efficacy of LP-80 in a nonhuman primate model. Five rhesus monkeys (K1 to K5) were intravenously infected with SHIV_SF162P3_ over 6 months, by which time chronic infection had been stably established. The set point viral loads ranged from 4.17 to 5.38 log_10_ RNA copies/ml after 191 days of infection (Fig 6). Then the monkeys were subcutaneously treated with LP-80 for two rounds. First, LP-80 was injected at 3 mg/kg of body weight once daily for 2 weeks. As expected, the plasma viral loads in three monkeys (K1, K2, and K4) precipitated below the assay detection limit (100 copies/ml) at day 4 after the initiation of treatment, which was the first blood sampling time, while the plasma viral loads in other two monkeys (K3 and K5) declined to below the detection limit 8 days after treatment, which was the second blood sampling time. To observe its last-acting activity, the same dose of LP-80 was administrated once every 4 days for 4 weeks. Encouragingly, the viral replication was fully controlled during the treatment period. As expected, the viral loads rebounded in all of the treated monkeys between 10 to 21 days after LP-80 was stopped.

After a 3-month interruption of the treatment, the monkeys had been chronically infected with SHIV_SF162P3_ for 325 days and maintained a mean plasma viral load of 4.5 log_10_ RNA copies/ml (ranging from 3.87 to 5.1). A second round treatment was initiated to observe the efficacy of LP-80 in a long-term monotherapy, in which the LP-80 was subcutaneously used at 3 mg/kg twice weekly for 6 months. As shown in Fig 6, one injection resulted in the viral loads below the detection limit in all of the five monkeys, which were determined at day 4 after the initiation of treatment, and no viral rebound was detected during a 6-month treatment. After LP-80 cessation, the virus rebounded in four monkeys (K1 to K4) between 14 to 28 days; however, the viral load in the monkey K5 remained undetectable even after LP-80 was withdrawn 2 months.

**Fig 7:**
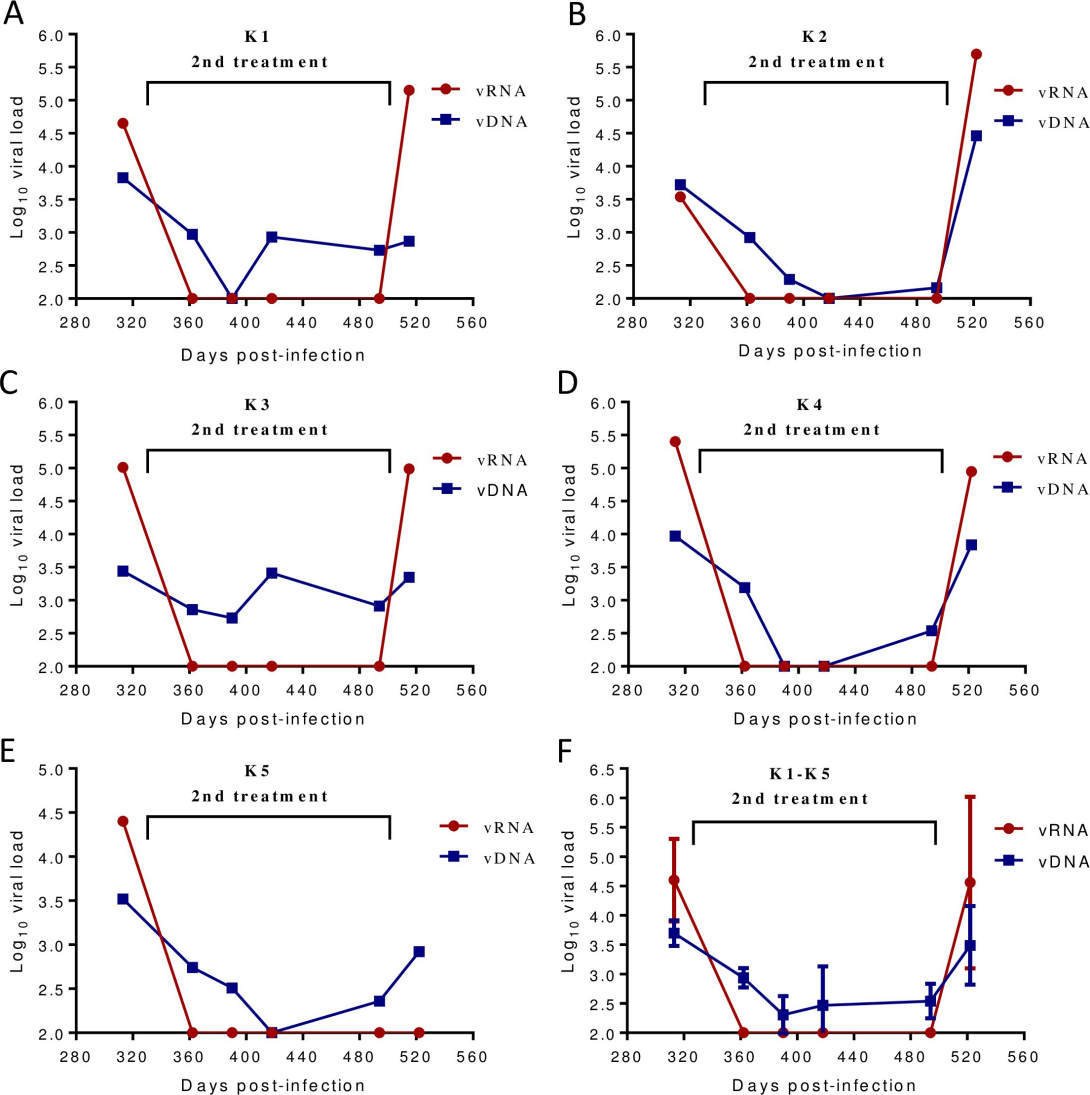
Changes of viral DNA and RNA loads in the monkeys during the 2^nd^ treatment. The viral DNA (vDNA) in the PBMC samples of the monkeys was measured by quantitative PCR. The correlation between the PBMC vDNA load (copies/μg total DNA) and plasma vRNA load (copies/ml) was analyzed by Pearson Correlation Coefficient: **(A)** monkey K1 (R2 = 0.3065, *P* = 0.2544); **(B)** monkey K2 (R2 = 0.8588, *P* = 0.0079); **(C)** monkey K3 (R2 = 0.4624, *P* = 0.1372); **(D)** monkey K4 (R2 = 0.7506, *P* = 0.0256); **(E)** monkey K5 (R2 = 0.6299, *P* = 0.0595); **(F)** monkeys K1-K5 (R2 = 0.8587, *P* = 0.0079).

We also detected viral DNA (vDNA) levels in the PBMC samples of monkeys pre- and posttreatment. It was found that a mean vDNA load was rapidly declined from 3.7 to 2.3 log_10_ vDNA copies/μg total DNA after a 2-month treatment and sustained a low level during the treatment (Fig 7). Noticeably, the vDNA loads in four monkeys (K1, K2, K4, and K5) could reach undetectable levels with a detection limit of 100 copies/μg total DNA. Similar to the plasma vRNA, the PBMC vDNA rebounded in all of the monkeys after LP-80 was stopped. As analyzed by Pearson Correlation Coefficient, the mean vDNA and vRNA loads were significantly correlated (R2 = 0.8587, *P* = 0.0079). During the treatment, LP-80 did not cause significant injection site reactions (ISRs). No systemic toxicities were observed in the monkeys by monitoring their blood biochemical parameters and CD4+ and CD8+ T lymphocytes (S1 Table and S1 Fig). Taken together, the results demonstrate that LP-80 exhibits an extremely potent and long-acting therapeutic efficacy and low cytotoxicity in SHIV-infected rhesus monkeys.

## Discussion

HIV infection requires membrane fusion between the virus and target cells, which is mediated by viral envelope (Env) glycoproteins gp120 and gp41 [24]. Binding of gp120 to cell receptors induces series conformational changes in Env complex and activates the fusogenic activity of gp41. In a current model, the N-terminal fusion peptide of gp41 is firstly inserted into target cell membrane; then its C-terminal heptad repeat (CHR) folds antiparallelly into the interior hydrophobic grooves adopted by the N-terminal heptad repeat (NHR) coiled coils, leading a stable six-helix bundle (6-HB) structure that drives membrane merger. The CHR-derived peptides, such as T-20, can competitively bind to viral NHR thus blocking 6-HB formation and viral entry [25]. In the past decade, we have dedicated our efforts to define the structure and function of gp41 and to develop new HIV fusion inhibitors. As results, we identified several structural features critical for the functionality of gp41, including the interhelical salt-bridges [26,27], M-T hook structure [28–30], and pocket-2 conformation [31]; we designed a group of inhibitors with significantly improved pharmaceutical profiles, including CP32M [32], HP23 [33], 2P23 [34], LP-11 [9], LP-19 [10], LP-46 [35], LP-50 and LP-51 [18], and LP-52 [17]. We also focused on selecting HIV mutants resistant to fusion inhibitors and proposed several resistance modes [36–40]. Meanwhile, a large panel of crystal structures were determined for diverse fusion inhibitors, including CP32 [29], CP32M [41], SFT [42], MT-C34 [28], MT-SFT [43], SC22EK and MTSC22EK [44], SC29EK [37], HP23L and LP-11 [45], LP-40 [16], LP-46 [35], and very recently, T-20 [46]. Combined, our series data have provided important information for understanding the mechanisms of HIV gp41-dependent membrane fusion and facilitated our development for the current fusion inhibitors that exhibit extremely potent and long-acting anti-HIV activity, as exemplified by LP-80 and LP-90.

Although T-20 was discovered in the early 1990s and approved for clinical use in 2003 [3,47–49], its mechanism of action and structural property remain elusive. For example, the inhibitory mode of T-20 was suggested to target the NHR helices, the CHR helices, the fusion peptide, and the transmembrane domain of gp41 and the coreceptor binding site of gp120 [15,47,48,50–56]. Therefore, we revisited T-20 by characterizing its structural and functional characteristics [16,46]. The crystal structures of T-20 and its lipopeptide derivative LP-40 did provide a molecular basis for elucidating the mode of action of T-20 and guide our inhibitor design. By integrating the strategies of sequence optimization and lipid conjugation, T-20 sequence-based lipopeptide derivatives exhibited extremely potent anti-HIV activity, with mean IC_50_ values in the very low picomolar range [17,18]. We recently reported the therapeutic efficacies of LP-50 and LP-51: they could sharply reduce viral loads to below the limitation of detection in acutely and chronically SHIV infected rhesus monkeys [18]. However, previous studies demonstrated the importance of fatty acid length, polarity, and bulkiness in improving the pharmacokinetics of peptide inhibitors [12,13,19,20]. Especially, we were inspired by success of the long-acting glucagon-like peptide-1 derivative semaglutide [21]. Thus, we focused on examining the effects of different fatty acids in the T-20 derivatives. As discovered, the fatty acid length does play crucial roles in the secondary structures of newly-designed HIV fusion inhibitors as well as their binding and inhibitory functions. As compared to C16-conjugated LP-52 and C18-conjugated LP-80, C12-conjugated LP-81 and C8-conjugated LP-82 had an identical peptide sequence but they showed dramatically reduced activities. Unexpectedly, the fatty acids with a further extended length (C20, C22, and C24) attenuated the anti-HIV ability of inhibitors, reflecting the complexity of the structure-activity relationship (SAR) of such lipopeptide-based fusion inhibitors.

Antiretroviral therapy (ART) has been very successful in treating HIV infection, but it does not eradicate the virus. Individuals infected with HIV require lifelong treatment with multiple drugs, which ultimately causes severe adverse effects and drug resistance. Also, noncompliance to the daily drug regimen often results in failure of the treatment regimen. As the only viral membrane fusion inhibitor available for clinical use, T-20 has shown effectivity in combination therapy of HIV-1 infection; however, it requires frequent injections at a high dosage and easily induces drug resistance, which have largely limited its wide application. From these perspectives, the most exciting result for LP-80 is its extremely high, long-acting antiviral activity. As demonstrated by the *in vivo* study, twice-weekly monotherapy with low-dose LP-80 could efficiently suppress the viral replication to below the limitation of detection during a 6-month treatment protocol. Consistently, the pharmacokinetics of LP-80 in rhesus monkeys had approved its long-acting activity, which even suggested a dosage once-weekly. By contrast, our previous studies demonstrated that C16-modified lipopeptides, such as LP-11, LP-19, LP-50, and LP-51, have shorter *in vivo* half-lives and thus require to be used once-daily [9,10,18]. Herein, we would like highlight the significance of two additional results. First, as one monkey remained aviremic following treatment cessation, it is valuable to investigate the potential of LP-80 for functional cure of HIV infection. Second, as the viral DNA level in the PBMCs of the treated monkeys markedly decreased, an early and/or long-term use of LP-80 might reduce the size of HIV reservoir. Furthermore, the extremely high genetic barrier to inducing drug resistance and therapeutic selectivity index characterized by LP-80 also make it an ideal drug for clinical use.

## Materials and methods

### Cells and plasmids

HEK293T cells and human T cell line MT-4 were purchased from American type culture collection (ATCC; Rockville, MD). The following reagents were obtained through the AIDS Reagent Program, Division of AIDS, NIAID, NIH: TZM-bl indicator cells, which stably express large amounts of CD4 and CCR5, along with endogenously expressed CXCR4, from John C. Kappes and Xiaoyun Wu; HL2/3 cells, which stably express high levels of Env, Gag, Tat, Rev, and Nef proteins of the integrated HIV-1 molecular clone HXB2/3gpt, from Barbara K. Felber and George N. Pavlakis; the Panel of Global HIV-1 Env Clones, which contains 12 envelope clones as reference strains representing the global AIDS epidemic, from David Montefiori; a panel of molecular clones for producing infectious HIV-1 isolates, including pNL4-3 from Malcolm Martin, pLAI.2 from Keith Peden, pSG3.1 from Sajal Ghosh, Beatrice Hahn, and George Shaw, pYK-JRCSF from Irvin SY Chen and Yoshio Koyanagi, p89.6 from Ronald G. Collman.

### Peptide synthesis and lipid conjugation

Peptides were synthesized on rink amide 4-methylbenzhydrylamine (MBHA) resin using a standard solid-phase 9-flurorenylmethoxycarbonyl (FMOC) method as described previously [9]. For fatty acid-based lipopeptides, the template peptide contain a lysine residue at the C-terminus with a 1-(4,4-dimethyl-2,6-dioxocyclohexylidene)ethyl (Dde) side-chain-protecting group, enabling the conjugation of a fatty acid that requires a deprotection step in a solution of 2% hydrazinehydrate-*N*,*N*-dimethylformamide (DMF). All peptides were acetylated at the N-terminus and amidated at the C-terminus. They were purified by reverse-phase high-performance liquid chromatography (HPLC) to more than 95% homogeneity and were characterized by mass spectrometry.

### The α-helicities and thermostabilities of lipopeptide inhibitors

The α-helicity and thermostability of lipopeptides in the absence or presence of a target mimic peptide (N39) were determined by circular dichroism (CD) spectroscopy. CD spectra were acquired on a Jasco spectropolarimeter (model J-815) using a 1 nm bandwidth with a 1 nm step resolution from 195 to 270 nm at room temperature. Spectra were corrected by subtracting a solvent blank. The α-helical content was calculated from the CD signal by dividing the mean residue ellipticity [θ] at 222 nm by the value expected for 100% helix formation (-33,000 degree.cm^2^.dmol^-1^). Thermal denaturation was performed by monitoring the ellipticity change at 222 nm from 20°C to 98°C at a rate of 2°C/min, and *T*_*m*_ (melting temperature) was defined as the midpoint of the thermal unfolding transition.

### Inhibitory activities of lipopeptide inhibitors on divergent HIV-1 isolates

The inhibitory activities of inhibitors on HIV-1_HXB2_ Env-mediated cell-cell fusion were determined by a reporter gene assay based on the activation of an HIV LTR-driven luciferase cassette in TZM-bl cells (target) by HIV-1 tat from HL2/3 cells (effector). Briefly, 1 x 10^4^/well of TZM-bl cells were plated in 96-well plates and incubated at 37°C overnight. Then, 3 x 10^4^/well of HL2/3 cells were cocultured with target cells for 6 h at 37°C in the presence or absence of an inhibitor at graded concentrations. Luciferase activity was measured using luciferase assay reagents and a Luminescence Counter (Promega, Madison, Wisconsin, USA).

The inhibitory activities of inhibitors against HIV-1 pseudoviruses and replication-competent isolates were measured as described previously [17]. Briefly, HIV-1 pseudoviruses were generated via the cotransfection of HEK293T cells with an Env-expressing plasmid and a backbone plasmid that encodes Env-defective, luciferase-expressing HIV-1 genome (pSG3Δenv). Viral stocks of replication-competent HIV-1 isolates were generated by transfecting viral molecular clones into HEK293T cells. Culture supernatants were harvested at 48 h posttransfection, and 50% tissue culture infectious doses (TCID_50_) in TZM-bl cells were determined. Inhibitors were prepared in 3-fold dilutions, mixed with 100 TCID_50_ of a virus, and then incubated for 1 h at room temperature. The mixture was added to TZM-bl cells (10^4^/well), and the cells were incubated for additional 48 h at 37°C. Luciferase activity was measured as described above.

### Therapeutic efficacy of LP-80 in SHIV-infected rhesus monkeys

The *in vivo* therapeutic efficacy of LP-80 was evaluated in SHIV-infected rhesus monkeys as described previously [10,18]. Five adult Chinese rhesus macaques (K1 to K5) were screened to be negative for SIV, herpes B virus, and simian T-lymphotropic virus. A simian-human immunodeficiency virus (SHIV_SF162P3_) was expanded on macaque peripheral blood mononuclear cells (PBMCs), and the TCID_50_ was determined. Macaques were intravenously inoculated with 1,000 TCID_50_ of virus. When chronic SHIV infection had been stably established after 6 months, the monkeys were subcutaneously treated with LP-80, which was dissolved in double-distilled and deionized water at 20 mg/ml. In the first round of treatment, LP-80 was injected at 3 mg/kg of body weight once daily for 2 weeks, followed by 3 mg/kg once every four days for 4 weeks. After a 3-month interruption, a second round of treatment was initiated with LP-80 used at 3 mg/kg, twice weekly for 6 months. Plasma viral loads (vRNA) were determined by a quantitative real-time reverse transcription-PCR (qRT-PCR) assay with the limit of detection at 100 copy equivalents of RNA per ml of plasma. To measure vDNA, total DNA extracted from the monkey PBMCs was used as input for quantitative PCR assay, and the vDNA copy numbers were estimated by comparison to a pGEM-SIV gag477 standard curve. Primers and probe used for both vRNA and vDNA were gag91 forward (GCAGAGGAGGAAATTACCCAGTAC), gag91 reverse (CAATTTTACCCAGGCATTTAATGTT), and pSHIVgag91-1 (5’-(FAM)-ACCTGCCATTAAGCCCGA—(MGB)-3’). Triplicate test reactions were performed for each sample.

### Pharmacokinetics of LP-80 in rats and rhesus monkeys

To determine the pharmacokinetics of LP-80 in rats, LP-80 was subcutaneously or intravenously injected to six Sprague-Dawley rats with a dose of 6 mg/kg, serum samples of rats were harvested before injection (0 h) and after injection (5, 15, 30 min, and 1, 2, 4, 8, 24, 48, 72, 96, 120, 168, 216 h). To determine the pharmacokinetics of LP-80 in non-human primates, LP-80 was subcutaneously injected to six Chinese rhesus macaques at 3 mg/kg, serum samples of macaques were harvested before injection (0 h) and after injection (1, 2, 4, 6, 8, 12, 18, 24, 36, 48, 60, 72, 96, 120,144, 168 h). Serum concentrations of LP-80 were measured by liquid chromatography and mass spectroscopy (LC-MS/MS), and pharmacokinetic parameters were derived via non-compartmental modeling. A single-cycle infection assay was performed to determine the inhibitory activity of the monkey sera on HIV-1_NL4-3_ pseudovirus. The 50% effective concentration was defined as the fold serum dilution that inhibited 50% of virus infection.

### Cytotoxicity of LP-80

The cytotoxicity of LP-80 on TZM-bl, MT-4, HEK293T, and human PBMC was measured using a CellTiter 96 AQ_ueous_ One Solution cell proliferation assay (Promega). In brief, 50-μl volumes of LP-80 at graded concentrations were added to cells, which were seeded on a 96-well tissue culture plate (1 × 10^4^ cells per well). After incubation at 37°C for 2 days, 20 μl of CellTiter 96 AQ_ueous_ One solution reagent was pipetted into each well and further incubated at 37°C for 2 h. The absorbance was measured at 490 nm using a SpectraMax M5 microplate reader.

### Selection of LP-80-resistant HIV-1 mutants

The *in vitro* selection of LP-80 resistant HIV-1 mutants was conducted as described previously [39]. MT-4 cells were seeded at 1×10^4^/well in RPMI 1640 medium containing 10% FBS on 12-well plates. HIV-1_NL4-3_ was used to infect the cells in the presence or absence of a diluted inhibitor (LP-80 or T-20). Cells were incubated at 37°C with 5% CO_2_ until an extensive cytopathic effect was observed. Culture supernatants were harvested and used for next passage on fresh MT-4 cells with a 2-fold increase in inhibitor concentrations.

### Ethics statement

Human PBMCs for determining the cytotoxicity of LP-80 were obtained from a previously existing collection [17], which was provided by the Beijing Red Cross Blood Center. A healthy blood donor for the sample was anonymized. Protocols for the use of animals were approved by the Institutional Animal Care and Use Committee (IACUC) at the Institute of Laboratory Animal Science, Chinese Academy of Medical Sciences (No. ILAS-VL-2015-004). To ensure personnel safety and animal welfare, the study of animals was conducted in accordance with the recommendations in the *Guide for the Care and Use of Laboratory Animals* of the Institute of Laboratory Animal Science and the recommendations of the Weatherall report for the use of non-human primates in research (http://www.acmedsci.ac.uk/more/news/the-use-of-non-human-primates-in-research/). All monkeys were housed and fed in an Association for Assessment and Accreditation of Laboratory Animal Care (AAALAC)-accredited facility. All animals were anesthetized with ketamine hydrochloride (10 mg/kg) prior to the procedures. The experiments were performed in bio-safety level 3 laboratory.

## Supporting information

S1 Table Changes of blood biochemical parameters in LP-80-treatment monkeys. The serum samples of LP-80-treated monkeys were prepared according to a standard protocol. The blood biochemical parameters in the monkey sera were determined by Model 7100 Hitachi Automatic Biochemical Analyser.
(DOCX)

S1 Fig Kinetics of CD4+, CD8+ T cell counts and CD4+/CD8+ T cell ratio in LP-80-treated monkeys. **(A)** Kinetics of absolute CD4+ T cell counts for each monkey during days 191–557 after infection. **(B)** Kinetics of absolute CD8+ T cell counts for each monkey during days 191–557 after infection. **(C)** Kinetics of the CD4+/CD8+ T cell ratio for each monkey during days 191–557 after infection. Polychromatic flow cytometry was performed for phenotyping of T lymphocytes. In brief, the peripheral blood samples of monkeys were collected into ethylene diamine tetraacetic acid (EDTA) anticoagulant tubes and peripheral blood mononuclear cells (PBMCs) were isolated by density gradient centrifugation. One million PBMCs were stained with the monoclonal antibody CD3-PerCP, CD4-FITC or CD8-PE (BD Biosciences, San Jose, CA). After washing with cold flow wash buffer, the cells were fixed with 1% paraformaldehyde and subjected to flow cytometry analysis within 24 hours. Samples were acquired and analyzed on a BD LSRII flow cytometer with the FACS Diva Software (BD Biosciences). FACS data were evaluated by the FlowJo Version 8.7 Software (Tree Star, Ashland, USA). Peripheral blood CD4+ or CD8+ T cell counts were calculated by multiplying the percentage of CD3+ CD4+ or CD3+ CD8+ T lymphocytes by the total lymphocyte counts.
(TIF)
