# Supplementary material for: Monotherapy with a low-dose lipopeptide HIV fusion inhibitor maintains long-term viral suppression in rhesus macaques
Source: PLoS Pathog. 2019 Feb 4;15(2):e1007552. doi: 10.1371/journal.ppat.1007552 (PMC6375636; doi:10.1371/journal.ppat.1007552)
Supplement: S1 Table — The serum samples of LP-80-treated monkeys were prepared according to a standard protocol. The blood biochemical parameters in the monkey sera were determined by Model 7100 Hitachi Automatic Biochemical Analyser. (DOCX) [file ppat.1007552.s001.docx]

S1 Table

|  | **1st treatment** | | **2nd treatment** | |
| --- | --- | --- | --- | --- |
| **Item （Unit）** | **Before (DPI 191)** | **After (DPI 263)** | **Before (DPI 325)** | **After (DPI 508)** |
| ALT (U/L) | 16.4 ± 3.91 | 15.8 ± 3.27 | 12.8 ± 3.27 | 13.2 ± 2.86 |
| AST (U/L) | 32.4 ± 3.29 | 30.8 ± 3.27 | 27.2 ± 3.11 | 31.4 ± 4.45 |
| TP (g/L) | 74 ± 4.53 | 71.26 ± 1.76 | 70.76 ± 3.48 | 71.15 ± 2.86 |
| ALB (g/L) | 45.49 ± 6.56 | 41.78 ± 1.31 | 50.99 ± 2.61 | 44.88 ± 4.31 |
| GLOB (g/L) | 25.04 ± 4.96 | 29.77 ± 0.85 | 22.23 ± 1.91 | 24.01 ± 2.93 |
| TBIL (umol/L) | 6.11 ± 1.57 | 5.52 ± 0.82 | 5.92 ± 0.9 | 5.52 ± 0.9 |
| ALP (U/L) | 4.6 ± 1.52 | 4.2 ± 1.3 | 5.6 ± 1.14 | 7.4 ± 2.07 |
| GGT (U/L) | 7.4 ± 1.82 | 9.2 ± 3.83 | 9 ± 1.58 | 10.4 ± 1.95 |
| GLU (mmol/L) | 4.32 ± 0.32 | 5.02 ± 0.98 | 3.69 ± 0.62 | 3.74 ± 0.85 |
| UN (mmol/L) | 8.14 ± 2 | 8.38 ± 0.74 | 9.34 ± 1.26 | 7.99 ± 0.92 |
| CREA (umol/L) | 47.83 ± 6.76 | 61.09 ± 13.5 | 42.03 ± 5.95 | 44.09 ± 5.5 |
| UA (umol/L) | 3.08 ± 0.61 | 3.25 ± 0.54 | 2.54 ± 0.28 | 2.54 ± 0.73 |
| Ca (mmol/L) | 2.36 ± 0.11 | 2.4 ± 0.06 | 2.17 ± 0.12 | 2.19 ± 0.03 |
| P (mmol/L) | 1.22 ± 0.25 | 0.98 ± 0.07 | 1.74 ± 0.19 | 1.29 ± 0.24 |
| CHO (mmol/L) | 4.42 ± 0.19 | 3.90 ± 0.53 | 3.87 ± 0.44 | 4.02 ± 0.51 |
| TG (mmol/L) | 0.58 ± 0.14 | 0.51 ± 0.07 | 0.67 ± 0.13 | 0.62 ± 0.21 |
| HDL-C (mmol/L) | 2.46 ± 0.07 | 1.96 ± 0.26 | 2.46 ± 0.31 | 2.66 ± 0.45 |
| LDL-C (mmol/L) | 1.73 ± 0.44 | 1.56 ± 0.37 | 1.59 ± 0.2 | 1.32 ± 0.32 |
| CK (U/L) | 46.4 ± 9.81 | 56.6 ± 10.01 | 54.6 ± 10.74 | 55.4 ± 14.19 |
| LDH (U/L) | 290 ± 62.25 | 443.4 ± 85.91 | 247.4 ± 35.3 | 262.8 ± 48.26 |
| IgG (g/L) | 7.12 ± 0.72 | 8.6 ± 1.74 | 8.58 ± 1.46 | 8.52 ± 1.32 |
| IgA (g/L) | 0.93 ± 0.33 | 1.05 ± 0.12 | 0.76 ± 0.31 | 0.86 ± 0.18 |
| IgM (g/L) | 1.28 ± 0.6 | 1.12 ± 0.56 | 1.41 ± 0.29 | 1.49 ± 0.29 |
| A/G | 2.03 ± 0.48 | 1.50 ± 0.2 | 2.31 ± 0.23 | 2 ± 0.4 |
| NA (mmol/L) | 139.6 ± 1.95 | 139.6 ± 1.52 | 138.8 ± 0.84 | 140.2 ± 1.92 |
| K (mmol/L) | 4.62 ± 0.13 | 4.56 ± 0.18 | 4.7 ± 0.2 | 4.64 ± 0.26 |
| CL (mmol/L) | 108.8 ± 1.64 | 109.4 ± 1.52 | 109.2 ± 0.84 | 109.8 ± 1.92 |

DPI, days post infection.
