# Supplementary figures and images for: Monotherapy with a low-dose lipopeptide HIV fusion inhibitor maintains long-term viral suppression in rhesus macaques
Source: PLoS Pathog. 2019 Feb 4;15(2):e1007552. doi: 10.1371/journal.ppat.1007552 (PMC6375636; doi:10.1371/journal.ppat.1007552)

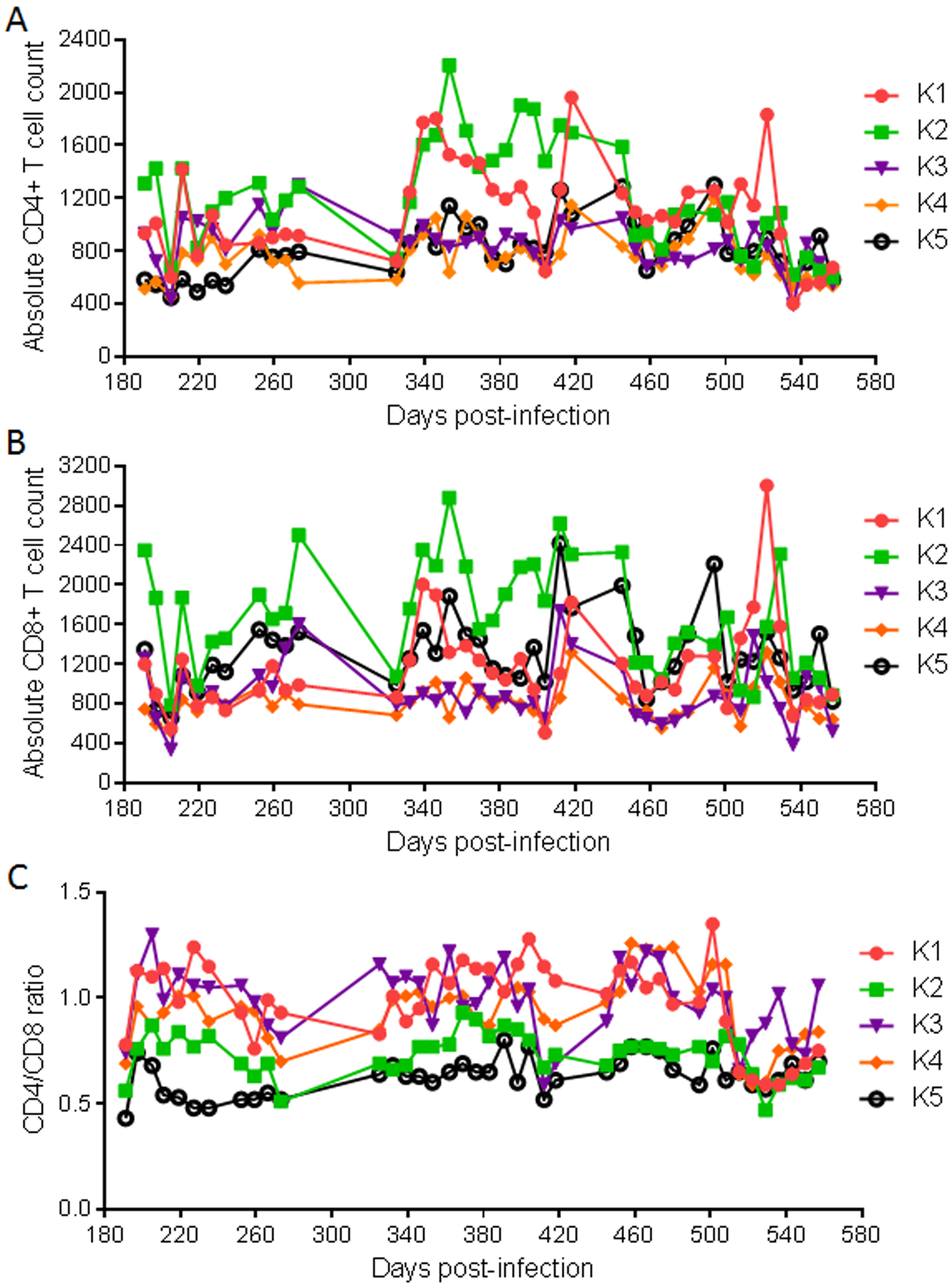

Supplement: S1 Fig — (A) Kinetics of absolute CD4+ T cell counts for each monkey during days 191–557 after infection. (B) Kinetics of absolute CD8+ T cell counts for each monkey during days 191–557 after infection. (C) Kinetics of the CD4+/CD8+ T cell ratio for each monkey during days 191–557 after infection. Polychromatic flow cytometry was performed for phenotyping of T lymphocytes. In brief, the peripheral blood samples of monkeys were collected into ethylene diamine tetraacetic acid (EDTA) anticoagulant tubes and peripheral blood mononuclear cells (PBMCs) were isolated by density gradient centrifugation. One million PBMCs were stained with the monoclonal antibody CD3-PerCP, CD4-FITC or CD8-PE (BD Biosciences, San Jose, CA). After washing with cold flow wash buffer, the cells were fixed with 1% paraformaldehyde and subjected to flow cytometry analysis within 24 hours. Samples were acquired and analyzed on a BD LSRII flow cytometer with the FACS Diva Software (BD Biosciences). FACS data were evaluated by the FlowJo Version 8.7 Software (Tree Star, Ashland, USA). Peripheral blood CD4+ or CD8+ T cell counts were calculated by multiplying the percentage of CD3+ CD4+ or CD3+ CD8+ T lymphocytes by the total lymphocyte counts. (TIF) [file ppat.1007552.s002.tif]
